# Bispectral index-guided anesthesia on time to tracheal extubation after onpump cardiac surgery

**DOI:** 10.1186/cc14573

**Published:** 2015-03-16

**Authors:** E Kaval, P Zeyneloglu, A Camkiran, A Sezgin, A Pirat, G Arslan

**Affiliations:** 1 Baskent University Faculty of Medicine, Ankara, Turkey

## Introduction

Electroencephalographic-based cerebral monitors such as the bispectral index (BIS) have been used for titration of both inhalational and intravenous anesthetics during general anesthesia. Titration of anesthetics using these monitors may facilitate an earlier recovery from general anesthesia and less consumption of anesthetics. The primary aim of this study was to investigate whether BIS-guided anesthesia would reduce time to tracheal extubation when compared with minimum alveolar concentration (MAC)-guided anesthesia in patients undergoing onpump cardiac surgery.

## Methods

Fifty patients undergoing elective coronary artery bypass grafting surgery from a single tertiary referral university hospital were randomized to BIS-guided anesthesia (Group BIS, *n *= 25) and MAC-guided anesthesia (Group MAC, *n *= 25). The inspired desflurane concentration was titrated to maintain a BIS value of 40 to 60 in Group BIS and an age adjusted minimum alveolar concentration of 0.7 to 1 was used in Group MAC. Time to tracheal extubation across the two groups was the primary outcome measure. Secondary outcomes included intraoperative desflurane consumption, postoperative complications, and lengths of stay in the ICU and hospital.

## Results

Demographic features, logistic EuroSCOREs, duration of cardiopulmonary bypass and surgery were similar in both groups. Mean desflurane consumption was significantly lower in Group BIS (11.9 ± 1.7 ml/hour) compared with Group MAC (13.4 ± 3.0 ml/hour) (*P *= 0.031). Time to tracheal extubation was not significantly different between the groups (13.3 ± 9.6 hours vs. 17.0 ± 22.4 hours) (*P *= 0.68). Incidences of postoperative complications were similar and lengths of stay in the ICU and hospital were 2.4 ± 0.7 days versus 3.2 ± 2.7 days and 5.3 ± 1.2 days versus 6.5 ± 3.1 days in Group BIS and Group MAC respectively (*P >*0.05 for all).

## Conclusion

Intraoperative use of BIS monitoring in patients undergoing onpump cardiac surgery reduced desflurane requirement but BIS-guided anesthesia did not facilitate time to extubation and lengths of stay in the ICU and hospital.

